# Lipidomic and Proteomic Alterations Induced by Even and Odd Medium-Chain Fatty Acids on Fibroblasts of Long-Chain Fatty Acid Oxidation Disorders

**DOI:** 10.3390/ijms221910556

**Published:** 2021-09-29

**Authors:** Khaled I. Alatibi, Stefan Tholen, Zeinab Wehbe, Judith Hagenbuchner, Daniela Karall, Michael J. Ausserlechner, Oliver Schilling, Sarah C. Grünert, Jerry Vockley, Sara Tucci

**Affiliations:** 1 Department of General Pediatrics, Adolescent Medicine and Neonatology, Faculty of Medicine, Medical Centre-University of Freiburg, 79106 Freiburg, Germany; Khaled.ibrahim.alatibi@uniklinik-freiburg.de (K.I.A.); sarah.gruenert@uniklinik-freiburg.de (S.C.G.); 2 Faculty of Biology, University of Freiburg, 79104 Freiburg, Germany; 3 Institute for Molecular Medicine and Cell Research, Faculty of Medicine, University of Freiburg, 79104 Freiburg, Germany; Stefan.tholen@uniklinik-freiburg.de (S.T.); oliver.schilling@uniklinik-freiburg.de (O.S.); 4 Institute of Surgical Pathology, Medical Center, University of Freiburg, 79104 Freiburg, Germany; 5 Department of Pediatric Hematology and Oncology, Center of Pediatric and Adolescent Medicine–Medical Center—University of Freiburg, Faculty of Medicine, 79106 Freiburg, Germany; Zeinab.wehbe@uniklinik-freiburg.de; 6 Department of Pediatrics II, Medical University Innsbruck, 6020 Innsbruck, Austria; Judith.Hagenbuchner@i-med.ac.at; 7 Department of Pediatrics I, Medical University Innsbruck, 6020 Innsbruck, Austria; Daniela.Karall@i-med.ac.at (D.K.); michael.j.ausserlechner@i-med.ac.at (M.J.A.); 8 School of Medicine, University of Pittsburgh, Pittsburgh, PA 15260, USA; vockleyg@upmc.edu; 9 Center for Rare Disease Therapy, UPMC Children’s Hospital of Pittsburgh, Pittsburgh, PA 15224, USA; 10 Graduate School of Public Health, University of Pittsburgh, Pittsburgh, PA 15260, USA

**Keywords:** lipidomics, proteomics, medium-chain fatty acids, long-chain fatty acid oxidation disorders, protein homeostasis, sphingolipid biosynthesis flux

## Abstract

Medium-chain fatty acids (mc-FAs) are currently applied in the treatment of long-chain fatty acid oxidation disorders (lc-FAOD) characterized by impaired β-oxidation. Here, we performed lipidomic and proteomic analysis in fibroblasts from patients with very long-chain acyl-CoA dehydrogenase (VLCADD) and long-chain 3-hydroxyacyl-CoA dehydrogenase (LCHADD) deficiencies after incubation with heptanoate (C7) and octanoate (C8). Defects of β-oxidation induced striking proteomic alterations, whereas the effect of treatment with mc-FAs was minor. However, mc-FAs induced a remodeling of complex lipids. Especially C7 appeared to act protectively by restoring sphingolipid biosynthesis flux and improving the observed dysregulation of protein homeostasis in LCHADD under control conditions.

## 1. Introduction

Long-chain fatty acid oxidation disorders (lc-FAOD) are monogenic inherited diseases affecting the mitochondrial β-oxidation of long-chain fatty acids (lc-FA). Lc-FAOD can be easily diagnosed though newborn screening (NBS) due to the accumulation of disease-specific acylcarnitine species that can be identified via tandem mass spectrometry [1,2]. Especially during situations of increased energy demand, when the organism mostly relies on β-oxidation for energy production, lc-FA cannot enter the β-oxidation cycle resulting in severe energy deficiency and in the accumulation of toxic metabolites. Therefore, treatment recommendations point at preventing catabolism and include avoidance of fasting, a fat-restricted diet and the application of medium-chain fatty acid (MCT oil) [3,4]. Recently, also the use of triheptanoin, a triglyceride with three molecules of heptanoic acid, was approved by the FDA for the treatment of lc-FAODs [5]. Both compounds contain either even or odd medium-chain fatty acids (mc-FAs) which are able to bypass the bottleneck represented by defective enzymes degrading lc-FA and can be fully metabolized, supplying organs and tissues with the required energy [6,7]. Indeed, the degradation of mc-FAs is mediated by different enzymes with substrate specificity for shorter fatty acid chains (C6-C12). Moreover, after chain shortening, the generated acyl-CoA can be redirected to the biosynthesis of ketone bodies available for energy production. In addition, heptanoic acid shows also a high anaplerotic potential by re-filling of citric acid cycle intermediates and provides substrates for gluconeogenesis [8,9,10,11,12].

A previous study has shown that impairment of mitochondrial fatty acid degradation due to lc-FAOD does not only result in the accumulation of toxic acylcarnitines but induces a systemic alteration of complex lipids that may contribute to the disease-specific phenotype. In keeping with the role of the α-subunit of the trifunctional protein and the very long-chain acyl-CoA dehydrogenase deficiency (VLCADD) in cardiolipin remodeling to ensure proper membrane binding and efficient FAO flux [13,14,15,16], the content and composition of mitochondrial cardiolipins was altered in VLCADD and long chain 3-hydroxyacyl-CoA dehydrogenase deficiency (LCHADD). This was associated with increased levels of proteins of mitochondrial respiratory complexes, likely as an adaptive/compensatory mechanism to maintain mitochondrial function despite defective β-oxidation [17,18].

Remodeling of acyl-residues in complex lipids and alteration of sphingomyelin biosynthesis fluxes towards ceramides from sphingomyelins was also observed in LCHADD, which suggested that the increased hexosylceramides may represent a possible contributing factor in the development of peripheral neuropathy in LCHADD [19]. In a similar manner, the increment of this lipid class has been described in several neurodegenerative diseases such as multiple sclerosis [20], Parkinson’s disease and macular degeneration [21,22,23].

In order to investigate whether incubation with mc-FAs alters the cellular lipidome thereby affecting cellular functions, we performed a comprehensive undirected lipidomic analysis as well as tandem mass tag-based (TMT) proteomic analysis in fibroblasts from patients with different lc-FAOD, namely, VLCADD and LCHADD, either after incubation with heptanoate (C7) or octanoate (C8). Our data suggest that treatment with mc-FAs does not only act in providing the required energy but also induces changes of the composition of complex lipids as well of the proteomic profile, which may be protective and improve symptoms.

## 2. Results

### 2.1. Even and Odd Medium Chain-Fatty Acids (mc-FAs) Induce Different Change in the Cellular Lipidome of Long-Chain Fatty Acid Oxidation Disorders (lc-FAOD)

**Figure 1:**
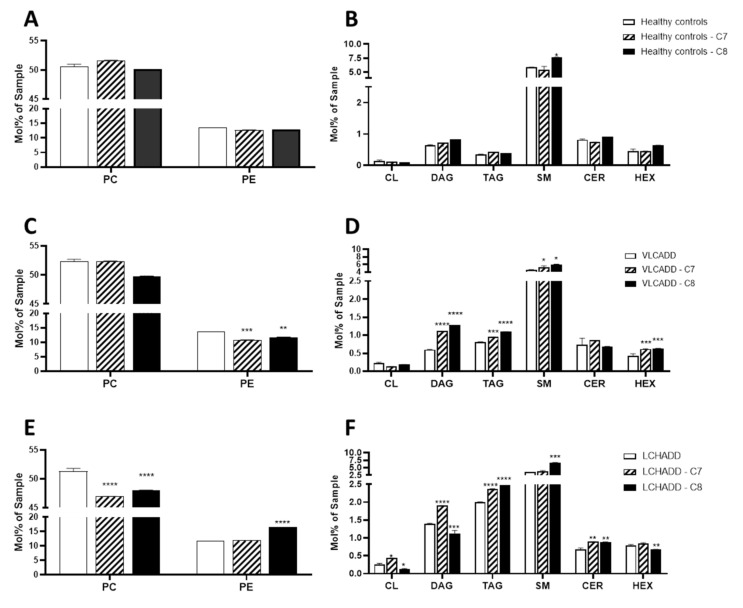
Effect medium-chain fatty acids on the lipidome of fibroblasts from healthy controls (**A**,**B**), VLCADD (**C**,**D**) and LCHADD (**E**,**F**). Mol% sample: indicates the moles of the lipid species extracted from one sample calculated as percentage of the total amount of lipid extract. PC: phosphatidylcholine; PE: phosphatidylethanolamine; SM: sphingomyelins; CL: cardiolipins; DAG: diacylglycerides; TAG: triacylglycerides; CER: ceramides; HEX: hexosylceramides; values denoted by * were considered significant if *p* < 0.05, ** *p* < 0.005, *** *p* < 0.0005, **** *p* < 0.0001 (two way ANOVA, genotype and diet were used as independent factors; Mann‒Whitney test; Friedman test and Tukey’s Test); * indicates significant differences between healthy controls and diseases. Lipid values under control conditions were retrieved from Alatibi et al. [19].

Previous studies in a mouse model of very long-chain acyl-CoA dehydrogenase deficiency (VLCAD^−/−^ mouse) have shown that supplementation with even and odd mc-FAs induces an alteration of the cellular fatty acid profile [11]. To investigate whether this effect occurred also in human fibroblasts from patients with lc-FAOD and whether these changes affect the metabolic response, we performed a comprehensive untargeted lipidomic analysis and proteomic analysis of fibroblasts from VLCADD and LCHADD patients either after incubation with C7 or C8. As depicted in Figure 1A,B, mc-FAs had little effect on the lipidome of fibroblasts from healthy controls with the exception of a significant increase of diacylglycerides (DAG) and sphingomyelin (SM) in cell lines after incubation with C8. In fibroblasts from VLCADD patients we observed a significant reduction of PC upon C8 and of PE after incubation with both mc-FAs (Figure 1C). In parallel with these findings, after incubation with C7 we observed an increment in the molar ratio of PC/PE (4.83 vs. 3.85) (Table 1) compared to untreated cell lines, which may be indicative of an adaptive mechanism to reduce endoplasmic reticulum stress in response to increased available substrates for energy production [24]. In addition, the concentration of storage lipids DAG and triacylglycerides (TAG) as well as of SM and hexosylceramides (HEX) increased significantly after incubation of both mc-FAs (Figure 1D), whereas the total cardiolipin (CL) content did not change significantly. As shown in Figure 1E, the content of PC was significantly lower in LCHADD cell lines treated either with C7 or C8 compared to control conditions (46.9 mol% sample ± 10.1 and 48 mol% sample ± 9.2 vs. 51.3 mol% sample ± 14.1). On the other hand, although the content of PE was unaffected after incubation with C7, the content of PE increased significantly in cells treated with C8 compared to control conditions (16.6 mol% sample ± 0.3 vs. 11.8 mol% sample ± 0.4) (Figure 1E), leading to a negative change in the molar ratio of PC/PE in C8-treated LCHADD fibroblasts (2.91 vs. 4.33; Table 2), suggesting a deterioration of the metabolic efficiency and impairment of the electron transfer chain [20]. In line with these findings was the significant reduction of CL upon incubation with C8, whereas C7 led to a significant increase of this lipid species (Figure 1F). In addition, the significant increase of ceramides after incubation with C7 seemed to reverse the sphingolipid flux towards HEX, as reported recently in LCHADD fibroblasts under control conditions [19]. A similar effect was observed also in cell lines treated with C8, which were characterized by a remarkable increase of SM and ceramides (CER) accompanied by a reduction of HEX.

### 2.2. Gentotype and Not Mc-FAs Induce Disease Specific Proteome Aleration in Long-Chain Fatty Acid Oxidation Disorders (lc-FAOD)

**Figure 2:**
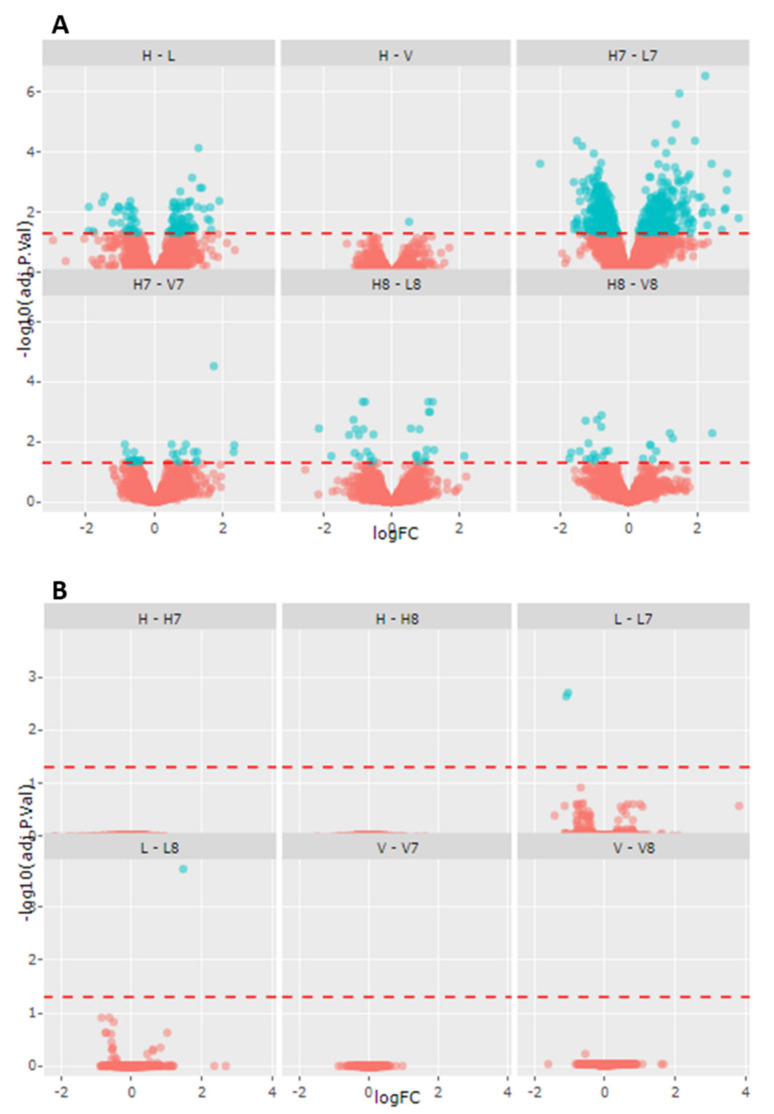
Volcano plots illustrating differently expressed proteins in fibroblasts of healthy controls (*n* = 3), VLCADD (*n* = 3) and LCHADD (*n* = 3) under control conditions and after incubation either with C7 or C8. (**A**) Genotype effect. (**B**) Treatment effect. H: healthy controls; V: VLCADD; L: LCHADD. H7-V7-L7: healthy controls, VLCADD and LCHADD after incubation with C7. H8-V8-L8: healthy controls, VLCADD and LCHADD after incubation with C8.

**Figure 3:**
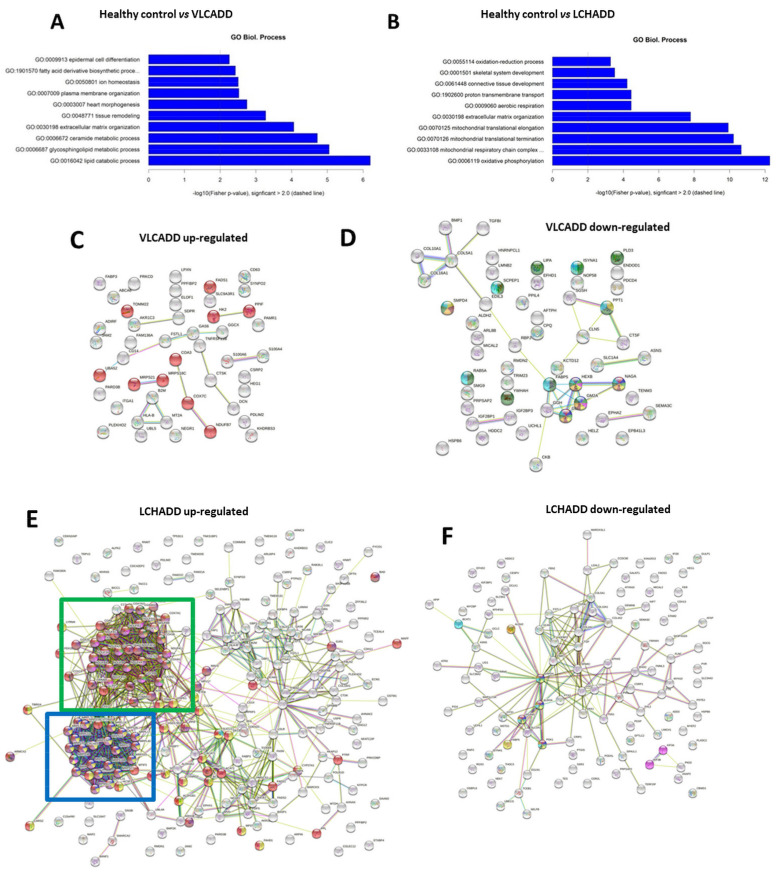
Disease-specific differently expressed proteins under control conditions. (**A**,**B**) Classification of the biological processes performed using the GO ontology enrichment software (http://geneontology.org/page/go-enrichment-analysis; accessed on 12 March 2021) [21,22]. (**C**,**D**) Up- and down-regulated VLCADD protein network identified via STRING software (http://www.string-db.org/; accessed on 12 May 2021). (**E**,**F**) Up- and down-regulated LCHADD protein network identified via STRING software (http://www.string-db.org/; accessed on 12 May 2021). A full list of the up- and down-regulated proteins is reported in Table S1.

Previous studies have already demonstrated impaired energy production in cell lines from LCHADD and VLCADD patients [18,19]. Our data showed a change in total CL content induced by mc-FAs, especially in LCHADD fibroblasts. As CL are essential for OXPHOS assembly and for the interaction of enzymes of mitochondrial β-oxidation with the mitochondrial supercomplexes of the respiratory chain [13,18], we performed proteomic analysis to investigate a possible proteome alteration secondary to changes of the cellular lipidome. Using the TMT-based proteomic approach, a total of 4651 proteins were identified and quantified. Comparisons were performed considering either the effect of the genotype or changes induced by the incubation with mc-FAs. The summary of the number of altered proteins between the groups is reported in Table 2 and Figure 2. Our findings clearly showed that the defective β-oxidation induces disease-specific adaptation/compensatory effects as reflected by the change in the proteome of fibroblasts with lc-FAOD (first six comparisons listed in Table 2). In contrast, the response of protein expression to treatment with mc-FAs was very moderate and only resulted in a few hits of significantly different expressed proteins (last six comparisons listed in Table 2 and Figure 2). In Figure 3 are represented the number of significantly altered proteins related to specific biological processes calculated using the GO ontology enrichment software (http://geneontology.org/page/go-enrichment-analysis; 20 March 2021) [25,26]. As clearly depicted in Figure 3A, in fibroblasts from VLCADD patients we could observe an alteration of biological processes involving metabolic pathways of complex lipids (Figure 3A). On the other hand, in fibroblasts from LCHADD patients we could confirm the altered processes of mitochondrial fusion and fission as well as a remarkable response of the mitochondrial respiratory system to LCHAD deficiency (Figure 3B), as previously described [17,18]. Of particular interest was the effect of C7 on healthy controls and LCHADD cell lines with a total of 922 differently regulated proteins (Table 2). Although under control conditions only 81 showed a significantly different expression compared to healthy controls, upon incubation with C7 the number of differently expressed proteins was 10-fold higher, indicative of a synergistic effect of genotype and treatment in this cell line (Table 2). With special regard to the effect of genotype, clustering of differently regulated proteins based on their metabolic pathway using STRING analysis (https://string-db.org/; accessed on 12 May 2021) revealed disease-specific adaptation of the proteome. As depicted in Figure 3C, VLCADD fibroblasts showed an up-regulation of proteins involved in remodeling of the cellular membrane. Although no functional clustering could be observed, this finding occurred in parallel to the down-regulation of enzymes of the lipid biosynthesis pathway, as shown in Figure 3D, suggesting that VLCADD may induce secondary membrane-associated lipid remodeling. In contrast, we identified two important protein association clusters in LCHADD cell lines (Figure 3E), proteins of OXPHOS complex and assembly (network on the top) as well as ribosomal proteins (bottom). This is in accordance to previously reported data [17,18]. Different from VLCADD, we observed a downregulation of proteins involved in the remodeling of the cellular membrane in LCHADD fibroblasts (Figure 3F).

### 2.3. Mc-FAs Deeply Alter Composition and Desaturation Degree of Mitochondrial Cardiolipins

**Figure 4:**
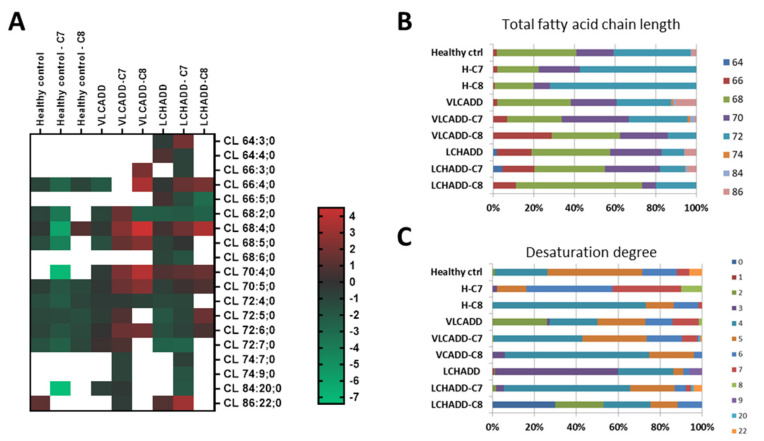
Disease-specific alteration of cardiolipins in healthy controls and VLCADD and LCHADD fibroblasts. (**A**) Heatmap of log2 transformed concentration of specific cardiolipin species. (**B**) Change of total fatty acid chain length. (**C**) Representation of the desaturation degree. Concentrations of the measured fatty acid chain length are represented as percentage. Mol% sample: indicates the moles of the lipid species extracted from one sample calculated as percentage of the total amount of lipid extract.

Because of the strikingly high number of differently expressed proteins in LCHADD fibroblasts treated with C7 compared to healthy controls (Table 2; 922 in total), we evaluated the effects of mc-FAs specifically on mitochondrial cardiolipins (CL). The heatmap of log2 transformed lipid concentrations from fibroblasts of healthy controls, VLCADD and LCHADD showed the CL composition after treatment with mc-FAs (Figure 4A). As depicted in Figure 4B, mc-FAs had a minor effect on fibroblasts from healthy controls; however, both C7 and C8 induced an increment of species with a chain length of 72 carbons, especially at the cost of species with shorter acyl residues (CL68 and CL70). While C7 had no remarkable effect on VLCADD fibroblasts, the composition of CL in VLCADD cell lines treated with C8 displayed a shift towards CL species with a chain length of 66 carbons (Figure 4B). Interestingly, also LCHADD fibroblasts showed a similar response to C8, as reflected by an increase of species with 68 carbons, while the effect of C7 treatment was minor (Figure 4B). In contrast, the analysis of the desaturation degree of the CL demonstrated that although the total fatty acid chain length was only moderately affected, the desaturation degree changed remarkably in VLCADD and LCHADD fibroblasts with an increment of species with a lower number of double bonds (Figure 4C), possibly affecting mitochondrial respiration performance. Indeed, alteration of mitochondrial lipid leaflet is reflected by physical changes of membrane fluidity with a strong subsequent impact on dynamics within the membranes [27,28].

### 2.4. Effect of Mc-FAs on Sphingolipid Metabolic Flow

**Figure 5:**
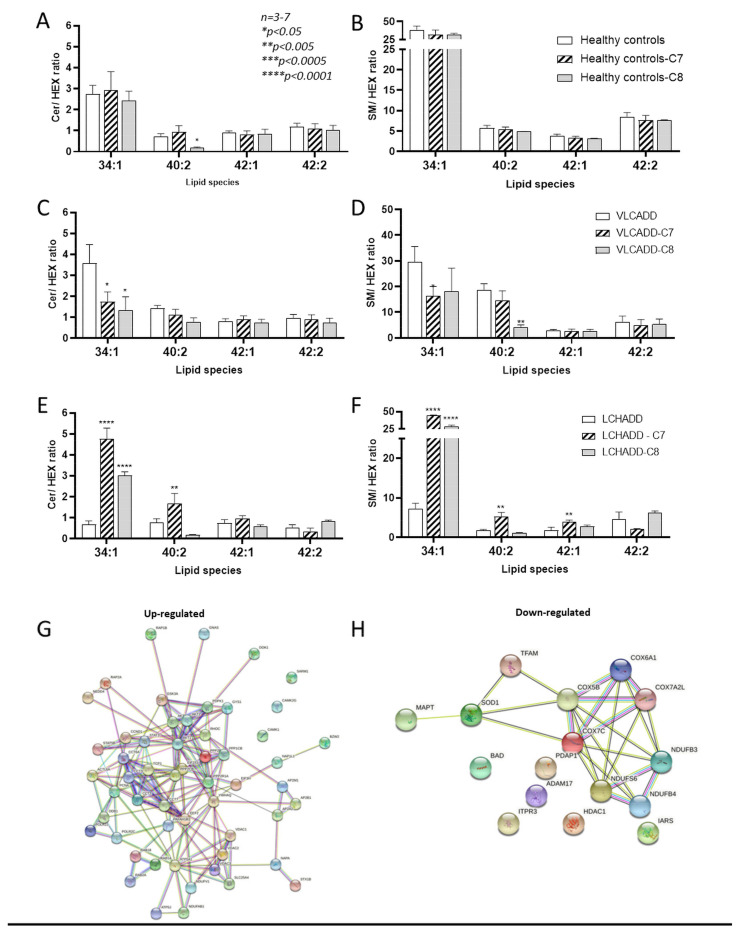
Effect of mc-FAs on ceramide/hexosylceramide and sphingomyelin/hexosylceramide ratios for 34:1, 40:2, 42:1 and 42:2 species. (**A**,**B**) Healthy controls; (**C,D**) VLCADD fibroblasts; (**E,F**) LCHADD fibroblasts. (**G**,**H**) Up- and down-regulated LCHADD protein network after incubation with C7 identified via STRING software (http://www.string-db.org/; 12 May 2021). Values denoted by * were considered significant if *p* < 0.05, ** *p* < 0.005, *** *p* < 0.0005, **** *p* < 0.0001 (two way ANOVA, genotype and diet were used as independent factors; Mann‒Whitney test, Friedman test and Tukey’s Test); * indicates significant differences between healthy controls and diseases.

Our previous data have shown a redirection of the sphingolipid metabolic flow towards the biosynthesis of hexosylceramides (Hex) in fibroblasts from LCHADD patients [19], and we hypothesized this alteration to be a possible contributing cause of the neuropathic phenotype in LCHADD and trifunctional protein deficiency (MTPD) [19,29]. We therefore investigated how mc-FAs further affect the sphingolipid metabolic flow. As depicted in Figure 5A,B, the ceramide/hexosylceramide (CER/HEX) and sphingomyelin/hexosylceramide (SM/HEX) ratio [30] for the specific species 34:1, 40:2, 42:1 and 42:2 did not change after incubation with mc-FAs in healthy controls. On the other hand, in VLCADD fibroblasts the CER/HEX ratio for the specific species 34:1 was significantly reduced upon incubation with both C7 and C8 (Figure 5C). In addition, C7 induced a slight but significant reduction of SM/HEX for the species 34:1, whereas C8 showed a similar effect on the species 40:2 (Figure 5D). In strong contrast, in LCHADD fibroblasts the application of mc-FAs redirected the sphingolipid metabolic flow towards the biosynthesis of SM, as shown in Figure 5E,F. Especially the incubation with C7 increased the CER/HEX and SM/HEX ratio to values comparable to those reported in healthy controls. Moreover, we could observe a striking increase up to 7-fold of the CER/HEX and SM/HEX ratio for the specific species 34:1. In particular, C7 resulted in a higher ratio also for the species 40:2 of CER/HEX and for 42:1 of SM/HEX (Figure 5E,F). It is therefore conceivable that C7 may improve neuropathic symptoms via remodeling of complex lipids [31,32,33,34]. On the other hand, C8 showed a similar effect on the ratio for the species 34:1; however, all other species remained unaffected (Figure 5E,F). Because of the remarkable effect of C7 on LCHADD fibroblasts, we had a closer look at the proteome after incubation with C7. Interestingly, we observed the highest response with a total of 922 significantly differently expressed proteins (Table 2) compared to healthy controls under the same conditions. In particular, KEGG analysis revealed an altered expression of proteins involved in neurodegenerative diseases such as amyotrophic lateral sclerosis and spinocerebellar ataxia (Figure S1). To obtain an overview on the effects of C7 on LCHADD fibroblasts, we considered only proteins that were significantly up- or down-regulated (*p*-value < 0.05) and we clustered them by their metabolic pathway using STRING analysis (Figure 5G,H). In line with the lipidomic data, using the TMT-based proteomic analysis we could demonstrate the up-regulation of the sphingolipid signaling pathway and ceramide biosynthesis also at protein level (PPP2CB,AKT3,PPP2R1A,PPP2R2A,PDPK1,AKT2,AKT1), accompanied by the up-regulation of proteins involved in mitochondrial respiration (Figure 5G). Of particular interest was the down-regulation of proteins specifically associated with neurodegenerative diseases such as the microtubule (MT)-associated protein tau (MAPT) [35], Cu/Zn superoxide dismutase (SOD1) [36,37] and the mitochondrial transcription factor A (TFAM) [38], as well as several components of the proteasome. These data suggested that C7 may reduce oxidative processes and restore lipid composition, contributing at least to inhibiting the progression of the symptoms in LCHADD.

## 3. Discussion

Long-chain fatty acid oxidation disorders (lc-FAOD) are a group of rare inborn errors of metabolism leading to severe energy deficits that may result in metabolic decompensation, especially during situations of increased energy demand [1]. Despite being monogenetic diseases, we have previously shown that functional impairment of one enzyme or enzyme complex of the mitochondrial β-oxidation with the subsequent accumulation of acyl-residues results in a systemic alteration of complex lipids that possibly also contribute to the chronic neuropathic phenotype seen in LCHADD [19]. In this work we show that even and odd medium-chain fatty acids (mc-FAs) such as C7 and C8, which are commonly applied in the treatment of lc-FAOD [3,4,5,32], do not only represent a source of energy but also induce disease- and treatment-specific alteration in the lipidomic and proteomic profile when they are supplemented to LCHADD and VLCADD fibroblasts.

Mc-FAs are generally absorbed more efficiently than long-chain fatty acids (lc-FA). Indeed, they reach the portal blood, avoiding the incorporation into chylomicrons mediated by enterocytes, and are transported to the liver [6]. Despite the clear advantage of being available for full degradation and energy supply, previous studies have also demonstrated the high potential for lipid remodeling in several tissues of VLCAD^−/−^ mice [11]. In fact, long-term supplementation with either MCT or triheptanoin indeed deeply altered the fatty acid profile of membrane lipids with a remarkable increase of saturated and monounsaturated lc-FA accompanied by a significant reduction of polyunsaturated species [11]. Our findings demonstrated that lipid remodeling also occurred in human fibroblasts of lc-FAOD after incubation with either C7 or C8 (see also Figure S2). Due to the importance of CL for several mitochondrial functions [39,40,41] and the fact that alteration in the composition is often associated with impaired mitochondrial metabolism [42,43,44,45,46,47], we mainly focused on the total content and composition of CL. The previously reported increase in CL content and altered CL composition in LCHADD fibroblasts [19] is supported by the finding of a remarkable up-regulation of components of oxidative phosphorylation (OXPHOS). This is likely a result of aberrant remodeling due to mutations in the *HADHA* gene in line with findings reported by Raimo et al. [18]. Of interest was the observation that both applied mc-FAs showed a comparable effect on CL remodeling by increasing the percentage of shorter total fatty acid chain lengths in either VLCADD or LCHADD fibroblasts. However, only C7 appeared to induce a clear metabolic response, as indicated by our proteomic analysis in LCHADD fibroblasts. This suggests that the change in CL composition accompanied by the supply of substrates from C7 for different mitochondrial pathways is able to improve mitochondrial metabolic efficiency and respiration and reduce the likely compensatory up-regulation of OXPHOS components.

PC and PE represent the major lipid class of all membranes, and along with CL, mitochondrial membrane is also very rich in PC and PE, whereby these metabolites account for up to 85% of the total phospholipid content [48]. The total PC and PE amount may differ to maintain organelle-specific functions as well as thickness and stiffness of the membranes. These two physical parameters specifically mediate transport and secretory efficiency of the organelles [49]. In this regard, the increased molar ratio of PC/PE upon supplementation with mc-FAs which occurred in parallel to a remarkable increment of DAG is of particular interest. These findings are supportive of a change in membrane structure under mc-FA treatment that may possibly have a beneficial effect on, e.g., neuropathic symptoms. In fact, the biophysical properties of the membranes are defined by the lamellar and spatial-temporal arrangement of lipid species and their composition [48]. In the supramolecular assembly the lipid species directly induce specific curvature in the cell membrane depending on the ongoing signaling and metabolic processes [50]. Despite the increment of DAG content, the concomitant reduction of PE could represent a protective cellular mechanism, as a parallel increase of both species DAG and PE induces regions of negative stress curvature in cellular membranes. These are associated with the deregulation of lipid/protein clustering leading to pathological states [51]. Although the applied mc-FAs differ in only one single carbon, in LCHADD fibroblasts the incubation with C8 resulted, surprisingly, in a remarkable reduction of the molar ratio of PC/PE accompanied by a reduction of DAG content. These data may suggest a triggered biosynthesis of SM and CER induced by the supplementation with C8. Indeed, PC is considered the phosphocholine moiety donor in the reaction of biosynthesis of CER, thereby generating DAG, which is a key intermediate in the biosynthesis of SM [52,53,54,55]. The supposed stimulation of the biosynthesis of SM was also confirmed by the evaluation of the CER/HEX and SM/HEX ratios after incubation with mc-FAs. We recently hypothesized that the redirection of the biosynthetic pathway towards higher HEX levels in LCHADD fibroblasts may contribute to the neuropathy in LCHADD and in mitochondrial trifunctional protein (MTPD) deficiency [19]. We here showed that incubation with both C7 and C8 was able to fully restore the CER/HEX and SM/HEX ratios to normal levels. After incubation with C8 this effect was limited to the specific species 34:1, while the reversion of sphingomyelin biosynthesis flux was more efficient after incubation with C7, as observed in most of the investigated CER/HEX and SM/HEX ratios. This finding is well in accordance with the up-regulation of proteins involved in the sphingolipid signaling pathway and ceramide biosynthesis measured via proteomic analysis. The therapeutic potential of C7 could therefore partly be attributed to the lipid remodeling of cellular membranes. Our data suggest that C7 might be more efficient than C8 in this respect. Peripheral neuropathy of LCHADD and MTPD is described as axonal neuropathy with possible secondary demyelination [56]. Our previous study demonstrated increased HEX levels in LCHADD fibroblasts [19]; if disruption of sphingolipid metabolic flux were to occur also in vivo, it would be indicative of a possible derangement of proper myelination, thereby affecting neurological function [57]. This effect was corroborated by the down-regulation of several proteins known to be associated with neurodegenerative diseases, such as Alzheimer’s (AD), Parkinson’s, Huntington’s and motor neuron diseases, as well as of proteasome components after incubation with C7 [35,36,37,38,58]. Dysregulation of protein homeostasis can be also taken into account for neurodegenerations due to the accumulation of misfolded and toxic proteins [58,59]. Indeed, lipid-assisted protein folding with the subsequent effects of protein functionality may be disrupted in case of alteration in the composition of lipid membranes [60,61,62]. In this case, the up-regulation of proteasome components as observed in LCHADD fibroblasts under control conditions may be indicative of an adaptive process to more efficiently digest misfolded proteins. We therefore hypothesize that the remodeling of lipid membranes upon C7 may reduce dysregulation of protein homeostasis in LCHADD fibroblasts more efficiently than after incubation with C8.

### Conclusions

In summary, we demonstrated that different defects of β-oxidation of lc-FA induced disease-specific alterations of the proteomic profile of fibroblasts compared to healthy controls. Incubation with mc-FAs had only minor effects in both healthy and lc-FAOD fibroblasts. Application with saturated mc-FAs resulted in an altered content and composition of membrane complex lipids. Although the changes upon both mc-FAs (C7 and C8) occurred to a similar extent, the effects of C7 were more pronounced, especially on LCHADD fibroblasts, where C7 appeared to be more beneficial. Indeed, in addition to supporting the mitochondrial metabolism, C7 was able to restore sphingolipid metabolic flux more efficiently than C8, reduced the expression of proteins known to be involved in neurodegenerative diseases and improved protein homeostasis. These findings were obtained in fibroblast cell lines of FAOD patients, which can obviously never allow full extrapolation to an in vivo situation. However, it is conceivable to suspect that similar effects may occur also in FAOD patients, as already confirmed in VLCAD*^-/-^* mice [11], although at different extent. We can therefore speculate that the positive therapeutic effects of MCT and triheptanoin applied in patients are due not only to energy supply but also to lipid remodeling as observed in fibroblasts. Further studies to elucidate the effects of C7 and C8 on complex lipids in lc-FAOD patients need to be performed.

## 4. Materials and Methods

### 4.1. Cell Culture

Experiments were performed with fibroblasts from four human healthy controls retrieved either commercially from the Coriell Institute (https://www.coriell.org/; accessed on 20 May 2021) or via biopsies from patients of Prof. Dr. Jerry Vockley (unpublished) and Prof. Dr. Daniela Karall [17]. Informed consent for research studies was obtained according to institutional guidelines. Detailed information on the cell lines is reported in Table 3. Skin dermal cells were grown in DMEM (×1) medium containing 10% fetal bovine serum (FBS), 4.5 g/l D-Glucose, GlutaMAX, 20 Mm HEPES, 100 units/mL penicillin and 100 μg/mL streptomycin, at 37 °C in air with 5% CO_2_. A severe form of VLCADD was confirmed in the used cell lines via enzyme testing with a method described previously [63]. All analyzed VLCADD cell lines showed residual activity in the range 0–10% compared to healthy controls. Incubation studies with either heptanoate (C7) or octanoate (C8) were performed, adding 300 µM of fatty acid to the control medium until the preparation of the pellets for the different analyses. To ensure a constant concentration of either C7 or C8, the medium was changed every second day.

### 4.2. Lipidomic Analysis

Mass spectrometry-based lipid analysis was performed by Lipotype GmbH (Dresden, Germany) as described [64]. Experiments were started with samples at passage 4 to 6 using the same initial cell density for each sample to synchronise the experimental condition. Fibroblasts were subcultured over 6 weeks either under control conditions or under incubation with either C7 or C8 until the needed amount of cells for analysis was reached. From this culture, 3 × 10^6^ cells were pelleted for lipidomic analysis and 5 × 10^6^ for further proteomics analysis or frozen when not needed. Lipids were extracted using a two-step chloroform/methanol procedure [65] and analysed as described previously [66,67,68,69].

### 4.3. Proteomic Analysis

Three to four biological replicates of each condition (9 conditions total; 33 samples) were used in a TMT-label-based proteome comparison. Pelleted cells were resuspended in lysis buffer (5% SDS, 50 mM triethyl ammonium bicarbonate (TEAB), pH 7.5) and sonicated using a Bioruptor device (Diagenode, Liège, Belgium). Samples were centrifuged at 13,000× *g* for 8 min and the supernatant used in the following steps. Proteins were reduced using 5 mM tris (2-carboxyethyl) phophine hydrochloride (TCEP) (Sigma, Taufkirchen, Germany) for 10 min at 95 °C and alkylated using 10 mM 2-iodoacetamide for 20 min at room temperature in the dark. The following steps were performed using S-Trap micro filters (Protifi, Huntington, NY, USA) following the manufacturer’s procedure. Briefly, a final concentration of 1.2% phosphoric acid and then six volumes of binding buffer (90% methanol; 100 mM triethylammonium bicarbonate, TEAB; pH 7.1) was added to 50 µg of protein. After gentle mixing, the protein solution was loaded to an S-Trap filter and spun at 2000 rpm for 0.5–1 min. The filter was washed three times using 150 μL of binding buffer. Sequencing-grade trypsin (Promega, Walldorf, Germany; 1:25 enzyme:protein ratio) diluted in 20 µL digestion buffer (50 mM TEAB) was added into the filter and digested at 47 °C for 1 h. To elute peptides, three step-wise buffers were applied: (a) 40 μL 50 mM TEAB, (b) 40 µL 0.2% formic acid in H_2_O and (c) 50% acetonitrile and 0.2% formic acid in H_2_O. The peptide solution was combined and dried in a SpeedVac. Peptides were resuspended in H_2_O and desalted using iST columns according to the manufacturers protocol (PreOmics, Martinsried, Germany) [70]. The peptide concentration was measured using BCA, and 25 µg of each sample was transferred to a fresh microreaction tube. Then, 0.15 M HEPES pH 8.0 was added. Samples were labeled using TMT-16-plex (Thermo Scientific, Waltham, MA, USA) [71]. Since only 16 samples can be compared in one 16 plex TMT panel, the 33 samples were divided in three 16 plex TMT panels, each panel containing replicates of every condition. For quantitative comparison of samples between different TMT panels, 2 channels of each TMT panel were used to measure a mastermix consisting of a small amount of every sample analyzed. In detail, 5 µg of every sample was mixed and 25 µg of this mixture used for labeling. Samples belonging to the same TMT panel were combined and 80 µg of each of the three TMT panels were fractionated via high pH reversed phase chromatography (XBridge C18 column, 150 mm  ×  1 mm column containing 3.5 µm particles (Waters)). An increasing linear gradient of acetonitrile from 10 to 45% over 45 min at a flowrate of 42 µL/min was applied using an Agilent 1100 HPLC system. A total of 36 fractions were collected and concatenated into 10 fractions, which were vacuum-concentrated until dryness and stored at −80 °C until LC–MS/MS analysis.

One microgram of peptides was analyzed on a Q-Exactive Plus mass spectrometer (Thermo Scientific, San Jose, CA, USA) coupled to an EASY-nLCTM 1000 UHPLC system (Thermo Scientific). The analytical column was self-packed with silica beads coated with C18 (Reprosil Pur C18-AQ, d = 3 Â) (Dr. Maisch HPLC GmbH, Ammerbusch, Germany). For peptide separation, a linear gradient of increasing buffer B (0.1% formic acid in 80% acetonitrile, Fluka) was applied, ranging from 5 to 40% buffer B over the first 90 min and from 40 to 100% buffer B in the subsequent 30 min (120 min separating gradient length). Peptides were analyzed in data dependent acquisition mode (DDA). Survey scans were performed at 70,000 resolution, and an AGC target of 3 × 10^6^ and a maximum injection time of 50 ms followed by targeting the top 10 precursor ions for fragmentation scans at 17,500 resolution with 1.6 m/z isolation windows, an NCE of 30 and an dynamic exclusion time of 35 s. For all MS2 scans the intensity threshold was set to 1.3 × 10^5^, the AGC to 1 × 10^4^ and the maximum injection time to 80 ms.

Raw data were analyzed with MaxQuant (v 1.6.14.0) (Max Plank Institute of Biochemistry, Martinsried, Germany) allowing two missed cleavage sites, no variable modifications and carbamidomethylation of cysteines as fixed modification; PIF was set to 0.75 and 16 plex TMT as isobaric label. The Human-EBI-reference database was downloaded from https://www.ebi.ac.uk/ (accessed on 20 January 2021). Only unique peptides were used for quantification.

Data were normalized on peptide level by equalizing the medians across all the channels and MS runs using the MSstatsTMT package (v. 1.8.2; Bioconductor, Buffalo NY) in R (v. 4.0.3; Vienna Austria). Subsequently, protein intensities were log2 transformed. To identify differentially expressed proteins, we used the limma package (v. 3.46.0; Vienna Austria) in R using the “robust” method. *p*-values were adjusted using the Benjamini‒Hochberg procedure (R: v. 4.0.3; Vienna Austria). Only proteins with an adjusted *p*-value  <  0.05 were considered as being significantly dysregulated.

### 4.4. Statistical Analysis

Data are presented as means ± standard deviation (SD). *n* denotes the number of biological replicates (*n* = 3–7; healthy controls *n* = 7; VLCADD *n* = 6; LCHADD *n* = 3). Statistical parametric and non-parametric analysis of lipidomics were conducted with LipotypeZoom Interactive Data and Visualisation software and with the Mann‒Whitney test and Friedman test using GraphPad Prism 6.0 (GraphPad Software, San Diego, CA, USA). The Mann‒Whitney test and Friedman test using GraphPad Prism 6.0 (GraphPad Software, San Diego, CA, USA) were applied to all other data. Differences were considered significant if *p* < 0.05. Statistical analysis of proteomic data is described above.

## Figures and Tables

**Table 1:** Phosphatidylcholine to phosphatidylethanolamine ratio measured in fibroblasts from healthy controls and from VLCADD and LCHADD patients after incubation with either C7 or C8.

	PC/PE Ratio		
	Control Conditions ^a^	C7	C8
Healthy controls	3.78	4.07	3.92
VLCADD	3.85	4.83 *	4.23
LCHADD	4.33	3.96	2.9 **

^a^ PE/PC values under control conditions were retrieved from Alatibi et al. [19]. Values denoted by * were considered significant if *p* < 0.05, ** *p* < 0.005.

**Table 2:** Number of altered proteins in fibroblasts from healthy controls and from VLCADD and LCHADD patients either under control conditions or after incubation with C7 or C8 detected by proteomic analysis.

Comparison	Number of Proteins Altered	Up-Regulated	Down-Regulated
Healthy control vs LCHADD	338	235	103
Healthy control vs VLCADD	104	48	56
Healthy control-C7 vs LCHADD-C7	922	406	516
Healthy control-C7 vs VLCADD-C8	232	146	86
Healthy control-C8 vs LCHADD-C8	231	115	116
Healthy control-C8 vs VLCADD-C8	312	90	122
Healthy control vs Healthy control-C7	36	5	31
Healthy control vs Healthy control-C8	7	7	
LCHADD vs LCHADD-C7	81	27	54
LCHADD vs LCHADD-C8	25	16	9
VLCADD vs VLCADD-C7	2	2	
VLCADD vs VLCADD-C8	9	5	4

**Table 3:** Detailed information on the cell line used in this study.

Disease	Origin	Sex	Allele 1	Allele 2
Healthy control	Coriell Institute (GM04501)	m	WT	WT
Healthy control	Coriell Institute (GM04505)	f	WT	WT
Healthy control	Coriell Institute (GM07492)	m	WT	WT
Healthy control	Coriell Institute (GM08399)	f	WT	WT
Healthy control	Coriell Institute (GM08400)	f	WT	WT
Healthy control	Coriell Institute (GM23964)	m	WT	WT
Healthy control	Coriell Institute (GM23976)	m	WT	WT
VLCAD	Coriell Institute (GM06127)	m	c.925G > a	c.925G > A
VLCAD	Coriell Institute (GM09093)	f	c.515T > C	c.637G > A
VLCAD	Coriell Institute (GM17475)	m	c.364A > G	c.364A > G
VLCAD	Prof. Dr. J. Vockley	f	c.1619T > C	c.1707_1715dupAGACGGGGC
VLCAD	Prof. Dr. J. Vockley	m	c.520G > A	c.1825G > A
VLCAD	Prof. Dr. J. Vockley	f	c.14T > C	c.1182 + 2dupT
LCHAD	Prof. Dr. D. Karall	m	c.1528G < C	c.1528G < C
LCHAD	Prof. Dr. D. Karall	f	c.1528G < C	c.1528G < C
LCHAD	Prof. Dr. D. Karall	m	c.1528G < C	c.1528G < C

M: male; f: female.

## Data Availability

The data presented in this study are available on request from the corresponding author. The data are not publicly available as they also contain original nonpublished data.

## Supplementary Materials

The following are available online at https://www.mdpi.com/article/10.3390/ijms221910556/s1, Figure S1: KEGG pathway of neurodegeneration (https://www.genome.jp/kegg/pathway.html). Figure S2: (A,B) Change of total fatty acid chain length and (C–H) Desaturation degree in PE and PC. Concentrations of the measured fatty acid chain length are represented as percentage. Table S1: List of proteins differentially regulated in LCHADD and VLCADD fibroblasts compared to healthy controls.
