# Supplementary material for: Lipidomic and Proteomic Alterations Induced by Even and Odd Medium-Chain Fatty Acids on Fibroblasts of Long-Chain Fatty Acid Oxidation Disorders
Source: Int J Mol Sci. 2021 Sep 29;22(19):10556. doi: 10.3390/ijms221910556 (PMC8508682; doi:10.3390/ijms221910556)
Supplement: Supplementary file 1 [file ijms-22-10556-s001.zip › Supplementary Figures.pptx]

## Slide 1
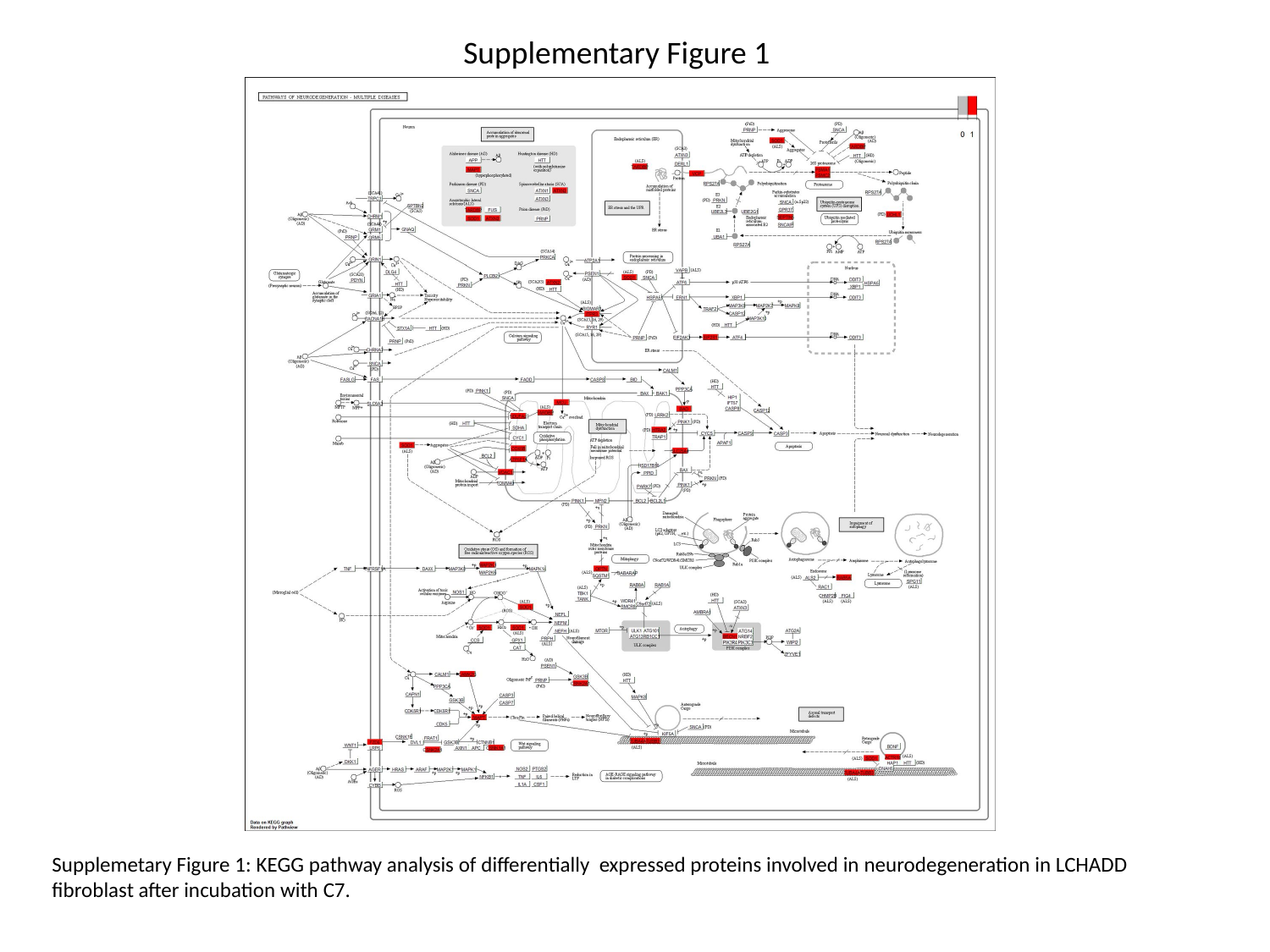

Supplementary Figure 1
Supplemetary Figure 1: KEGG pathway analysis of differentially expressed proteins involved in neurodegeneration in LCHADD fibroblast after incubation with C7.

## Slide 2
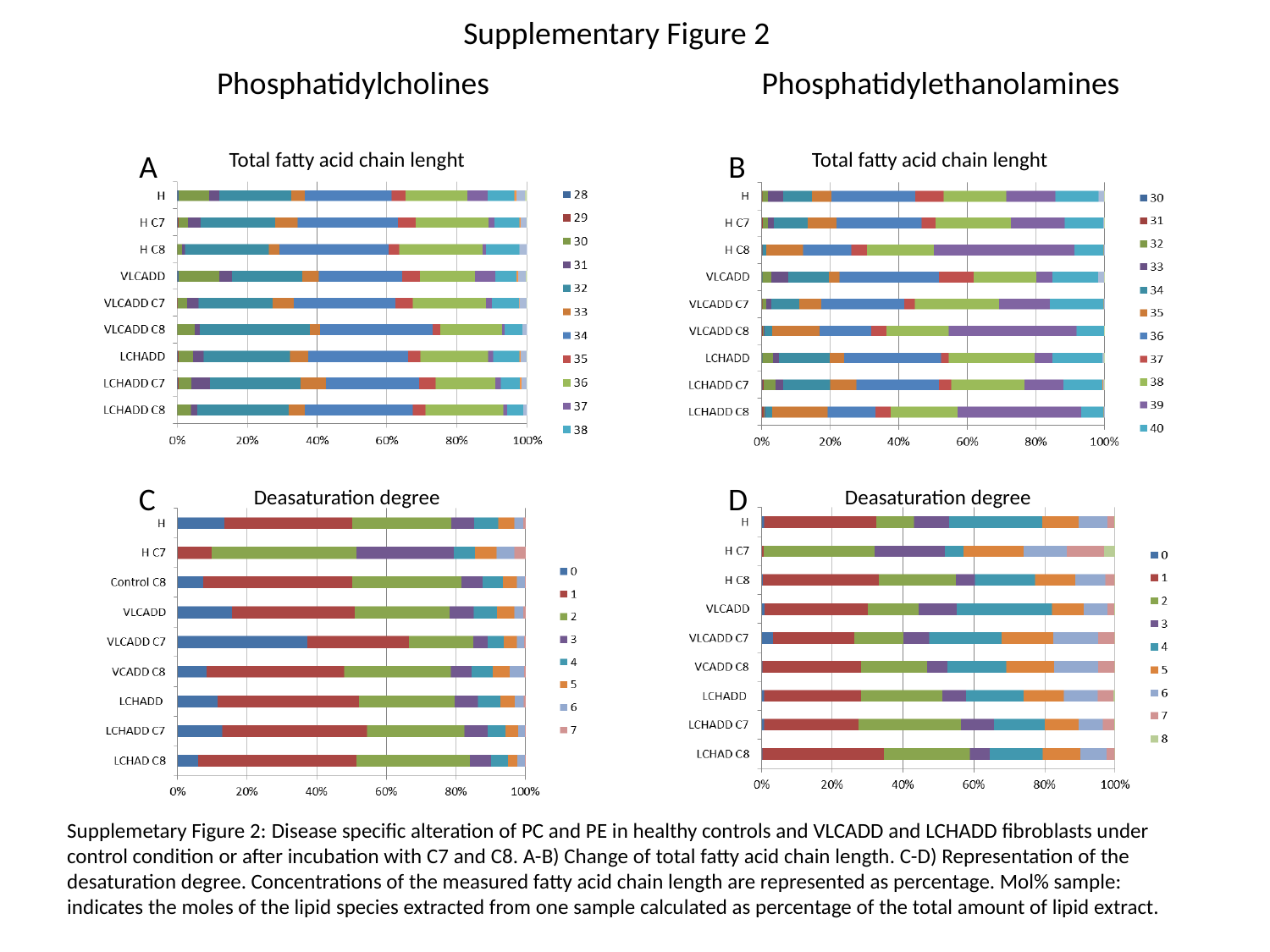

Supplementary Figure 2
Phosphatidylcholines
Phosphatidylethanolamines
A
Total fatty acid chain lenght
B
Total fatty acid chain lenght
C
D
Deasaturation degree
Deasaturation degree
Supplemetary Figure 2: Disease specific alteration of PC and PE in healthy controls and VLCADD and LCHADD fibroblasts under control condition or after incubation with C7 and C8. A-B) Change of total fatty acid chain length. C-D) Representation of the desaturation degree. Concentrations of the measured fatty acid chain length are represented as percentage. Mol% sample: indicates the moles of the lipid species extracted from one sample calculated as percentage of the total amount of lipid extract.
